# Characteristics of inflammatory reactions during development of liver abscess in hamsters inoculated with *Entamoeba nuttalli*

**DOI:** 10.1371/journal.pntd.0006216

**Published:** 2018-02-08

**Authors:** Yue Guan, Meng Feng, Xiangyang Min, Hang Zhou, Yongfeng Fu, Hiroshi Tachibana, Xunjia Cheng

**Affiliations:** 1 Department of Medical Microbiology and Parasitology, School of Basic Medical Sciences, Fudan University, Shanghai, China; 2 Department of Clinical Laboratory Medicine, Yangpu Hospital of Tongji University, Shanghai, China; 3 Department of Infectious Diseases, Tokai University School of Medicine, Isehara, Kanagawa, Japan; CINVESTAV, Mexico City, MEXICO

## Abstract

**Background:**

*Entamoeba nuttalli* is an intestinal protozoan with pathogenic potential that can cause amebic liver abscess. It is highly prevalent in wild and captive macaques. Recently, cysts were detected in a caretaker of nonhuman primates in a zoo, indicating that *E*. *nuttalli* may be a zoonotic pathogen. Therefore, it is important to evaluate the pathogenicity of *E*. *nuttalli* in detail and in comparison with that of *E*. *histolytica*.

**Methodology/Principal findings:**

Trophozoites of *E*. *nuttalli* GY4 and *E*. *histolytica* SAW755 strains were inoculated into liver of hamsters. Expression levels of proinflammatory factors of hamsters and virulence factors from *E*. *histolytica* and *E*. *nuttalli* were compared between the two parasites. Inoculations with trophozoites of *E*. *nuttalli* resulted in an average necrotic area of 24% in liver tissue in 7 days, whereas this area produced by *E*. *histolytica* was nearly 50%. Along with the mild liver tissue damage induced by *E*. *nuttalli*, expression levels of proinflammatory factors (TNF-α, IL-6 and IL-1β) and amebic virulence protein genes (lectins, cysteine proteases and amoeba pores) in local tissues were lower with *E*. *nuttalli* in comparison with *E*. *histolytica*. In addition, M2 type macrophages were increased in *E*. *nuttalli-*induced amebic liver abscesses in the late stage of disease progression and lysate of *E*. *nuttalli* trophozoites induced higher arginase expression than *E*. *histolytica in vitro*.

**Conclusions/Significance:**

The results show that differential secretion of amebic virulence proteins during *E*. *nuttalli* infection triggered lower levels of secretion of various cytokines and had an impact on polarization of macrophages towards a M1/M2 balance. However, regardless of the degree of macrophage polarization, there is unambiguous evidence of an intense acute inflammatory reaction in liver of hamsters after infection by both *Entamoeba* species.

## Author summary

*Entamoeba nuttalli* is the phylogenetically closest protozoan to *Entamoeba histolytica* and is highly prevalent in macaques. Previous studies have indicated that *E*. *nuttalli* is virulent in a hamster model. In this study, we compared the immunopathological basis of formation of liver abscess in hamsters between *E*. *nuttalli* and *E*. *histolytica*. Mild liver tissue damage developed after intrahepatic injection of trophozoites of *E*. *nuttalli*, and lower expression levels of genes for host proinflammatory factors and amebic virulence proteins were detected at the edges of liver abscesses induced by *E*. *nuttalli*. In addition, alternatively activated macrophages were increased in *E*. *nuttalli*-induced liver abscesses in the late stage of disease progression. The lysate of *E*. *nuttalli* trophozoites also induced higher arginase expression than *E*. *histolytica in vitro*. Polarization of macrophages is likely to affect the degree of acute inflammatory reactions in liver in an animal model during *E*. *nuttalli* infection. Our data reveal new characteristics of abscess formation by *E*. *nuttalli*.

## Introduction

The enteric protozoan *Entamoeba histolytica* causes an estimated 50 million cases of amebic colitis and liver abscess in humans, resulting in 40,000 to 100,000 deaths annually [1–5]. *Entamoeba dispar* is morphologically indistinguishable from *E*. *histolytica*, but is nonpathogenic. *E*. *histolytica* and *E*. *dispar* are also found in feces of nonhuman primates [6]. Recently, *Entamoeba nuttalli*, which is phylogenetically closer to *E*. *histolytica* than *E*. *dispar*, has also been identified in nonhuman primates [7], and there is a high prevalence of *E*. *nuttalli* infections in wild and captive macaques, including *Macaca mulatta*, *M*. *fasciculalis*, *M*. *fuscata*, *M*. *thibetana* and *M*. *sinica*, and other nonhuman primates in zoos [8–15]. Most macaques with *E*. *nuttalli* infections are asymptomatic, suggesting that the host-parasite relationship in macaques may be commensal in natural infection [12]. More recently, cysts of *E*. *nuttalli* were detected in a caretaker of nonhuman primates in a zoo [16]. The infected person was asymptomatic, but this finding raises the possibility that *E*. *nuttalli* is a zoonotic pathogen.

Fatal cases with liver abscess due to *E*. *nuttalli* have been reported in Abyssinian colobus and Geoffroy’s spider monkey in a zoo [17, 18], and inoculation of *E*. *nuttalli* trophozoites in liver of hamsters causes formation of abscesses and is lethal in some cases [7, 10, 12]. Hamsters inoculated with *E*. *nuttalli* are weakened and have decreased body weight. The liver lesions produced by *E*. *nuttalli* trophozoites are characterized by extensive necrosis associated with inflammatory reactions [7, 10]. These histological changes are similar to those caused by *E*. *histolytica* trophozoites, suggesting similar pathological mechanisms of tissue damage [7, 10, 19, 20]. However, *E*. *histolytica* infection in liver generally results in large single abscesses [1, 21], whereas *E*. *nuttalli* infection in hamsters induces small multiple abscesses [7]. Thus, the detailed mechanisms of how hosts with *E*. *nuttalli* develop a different pathogenic manifestation from that in *E*. *histolytica* infection are poorly defined.

*E*. *nuttalli* is as virulent as *E*. *histolytica* in animal models, but it remains unclear whether *E*. *nuttalli* is virulent in humans. These findings, coupled with *in vivo* observations that *E*. *nuttalli* causes histological lesions in similar conditions and has few sequence differences in some important genes [7, 14] in comparison with *E*. *histolytica*, have reinforced the idea that *E*. *nuttalli* is incapable of generating human lesions because of the host specificity of *E*. *nuttalli* and *E*. *histolytica* parasites. Therefore, it is important to evaluate the pathogenicity of *E*. *nuttalli* in comparison with that of *E*. *histolytica* to examine the molecular basis of the pathophysiology of amebic liver abscess (ALA) formation. In this study, expression levels of proinflamatory factors in hamsters and virulence factors from *E*. *histolytica* and *E*. *nuttalli* were compared between these parasites. The histopathological and immunopathological analyses of ALA provide valuable information on the pathogenicity of *E*. *nuttalli*.

## Methods

### Ethics statement

All animal experiments were performed in strict accordance with the Regulations for the Administration of Affairs Concerning Experimental Animals (1988.11.1) and were approved by the Institutional Animal Care and Use Committee (IACUC) of our institutions (Permit Numbers 20110307–051 and 20160225–097). All efforts were made to minimize suffering.

### Amebas and mammalian cells

Trophozoites of *E*. *histolytica* SAW755CR and *E*. *nuttalli* GY4 strains were grown under axenic conditions at 36.5°C in YIMDHA-S medium [22] containing 15% (v⁄v) heat-inactivated adult bovine serum. Trophozoites were harvested during the logarithmic growth phase (48 to 72 h) by chilling on ice for 5 min. RAW264.7 cells were cultured in DMEM (Thermo) supplemented with 10% fetal bovine serum (FBS) (Thermo), 100 U/ml penicillin, and 100 μg/ml streptomycin. CHO-K1 cells were cultured in Ham’s F12 nutrient medium (Thermo) supplemented with 10% FBS, 100 U/ml penicillin, and 100 μg/ml streptomycin. The mammalian cells were grown in a 37°C incubator with 5% CO_2_.

### Animal model for ALA

Six-week-old male hamsters were obtained from Shanghai Songlian Experimental Animal Factory. ALA was induced by direct inoculation of 1×10^6^ axenic trophozoites of *E*. *histolytica* SAW755CR strain or *E*. *nuttalli* GY4 strain into liver, as previously described [23].

### Pathologic evaluation using liver tissue sections

After intrahepatic inoculation of trophozoites, hamsters were euthanized at 3 h, 6 h, 12 h, 24 h, 48 h, 72 h and 168 h post-inoculation. At each time point, 6 to 7 hamsters were used. Liver tissues were harvested and fixed in 4% paraformaldehyde followed by paraffin embedding. Sections were stained with hematoxylin and eosin (HE) or periodic acid-Schiff (PAS) for histopathology [7]. Tissue damage and inflammatory cell infiltration were quantified in high quality images (2560×1920 pixels) captured using a Nikon light microscope. Areas of leukocyte infiltration and liver necrosis were measured using Image-Pro Plus 4.5.1 software (Media Cybernetics). Areas of interest are expressed as a percentage of the total tissue area.

### Analysis of cytokines by immunohistochemistry and quantitative real-time PCR

Immunohistochemical staining was performed as described elsewhere [24]. Briefly, paraformaldehyde-fixed liver sections were deparaffinized, rehydrated by standard protocols and incubated overnight at 4°C with rabbit anti mouse IFN-γ, TNF-α, IL-1β and IL-6 polyclonal antibodies (Abcam). The slides were subsequently incubated with horseradish peroxidase-labeled goat anti-rabbit immunoglobulin and then with chromogen substrate (3,3′-diaminobenzidine) for 2 min before counterstaining with hematoxylin. The cytokine score (intensity area/total image area) was determined in areas of leukocyte infiltration using Image-Pro Plus 4.5.1 software (Media Cybernetics). Each cytokine score was determined by counting more than 50 high-power fields (×20).

Gene expression of IFN-γ, TNF-α, IL-1β, IL-4, IL-6, IL-10, nitric oxide synthase (iNOS), arginine enzyme I (Arg-1) and mannose receptor I (MRC-I) in liver tissues was examined by quantitative real-time PCR (qRT-PCR) using the primers listed in Table 1 [22, 25–26]. Briefly, total RNA (1 μg) of tissue from the edge of a liver abscess was purified with an RNeasy Plus Mini kit (Qiagen). cDNA was synthesized with a Primescript first-strand cDNA synthesis kit (Takara) using oligo(dT) primers. qRT-PCR was carried out in a final reaction volume of 20 μl on an ABI 7500 Real-time PCR system (Applied Biosystems). Reactions were performed in a 96-well plate with SYBR Premix Ex Taq (Takara, Japan) containing primers listed in Table 1. The amplification cycling conditions were as follows: 30 s at 95°C and 40 cycles of 5 s at 95°C and 35 s at 60°C. Analysis by qRT-PCR of gene expression was conducted during the log phase of product accumulation, during which Ct values correlated linearly with relative DNA copy numbers. Each experiment was performed at least three times.

**Table 1:** Primers used in this study.

Hamster-IFN-γ	S	CCATCCAGAGGAGCATAG
	AS	CAGCACCGACTTCTTTTC
Hamster-TNF-α	S	CCTCCTGTCCGCCATCAAG
	AS	CACTGAGTCGGTCACCTTTC
Hamster-IL-6	S	AGGACACTACTCCCAACAG
	AS	GAGGCATCCATCATTTATT
Hamster-IL-1β	S	AATGCCTCGTGCTGTCTG
	AS	TTGTTGCTTGTTTCTCCCT
Hamster-IL-4	S	TGCACCGAGATGGTCGTAC
	AS	GTCTTTGAGAACCCTGGAAT
Hamster-IL-10	S	CAACTGCAGCGCTGTCATCGATTT
	AS	ATGGCCTTGAAGACGCCTTTCTCT
Hamster-iNOS	S	GCCTGCCGTAGCCAACAT
	AS	CAGAAGTCTCGAACGCCAAT
Hamster-Arg-1	S	GCCTTTGCTGATGTCCCT
	AS	GCTTCCAACTGCCATACTG
Hamster-MRC1	S	AGATAAACTCCAAGTCTGCCTTAA
	AS	ACTGCCAACCACTGCTGAAA
Hamster-β-actin	S	TCTACAACGAGCTGCG
	AS	CAATTTCCCTCTCGGC
Hgl	S	TGTGGTGGAGATTCTACA
	AS	CATCACCAACTGCTTGAA
Igl	S	AAATGTTCTTGTGGTGATG
	AS	GTGCACAACTTTTCTTCTT
CP2	S	ATCCAAGCACCAGAATCAGT
	AS	TTCCTTCAAGAGCTGCAAGT
CP5	S	AAAGAATGTTCATCAACTCAGCTT
	AS	TTAAGCATCAGCAACCCCAACTGG
AP-A	S	TAATCTTCGCTGTTGCTTT
	AS	TCAGCTCCCTTAGTGGTAA
AP-B	S	TGCTATTGCCTTTGCTGC
	AS	TTCGCAAACAACGACTGG
Eh-actin	S	GCACTTGTTGTAGATAATGGATCAGGAATG
	AS	ACCCATACCAGCCATAACTGAAACG

### Analysis of gene expression of amebic virulence proteins by qRT-PCR

Gene expression of heavy subunit of galactose/N-acetylgalactosamine lectin (Hgl), intermediate subunit of galactose/N-acetylgalactosamine lectin (Igl), cysteine proteinase 2 (CP2), cysteine proteinase 5 (CP5), amoebapore A (AP-A), and amoebapore B (AP-B) of *Entamoeba* trophozoites in ALA tissues were examined using the same RNA samples extracted from tissue at the edge of a liver abscess. Genes of virulence proteins of *E*. *nuttalli* GY4 strain were amplified and sequenced (S1 Text). Primers for these genes were designed using the identical sequence regions of *E*. *histolytica* SAW755CR strain and *E*. *nuttalli* GY4 strain, and are listed in Table 1. The primers for Hgl and Igl used in this study can amplify all known subtypes of Hgl and Igl genes. Reactions were performed as described above. Each experiment was performed at least three times.

### *In vitro* stimulation of macrophages by trophozoite lysates of amebas

To assess whether secretory proteins from *E*. *histolytica* and *E*. *nuttalli* cause polarization of macrophages, trophozoites were first incubated with CHO cells and then lysed. RAW264.7 cells were stimulated with the lysates of ameba trophozoites. Briefly, CHO-K1 cells (10^6^) were cultured in a 35-mm dish (Costar), and then 5×10^5^ trophozoites were added and coincubated for 30 min. Trophozoites were then harvested, and lysed by repeated freezing and thawing of 10^6^ trophozoites per ml in PBS. After centrifugation at 20,000 g for 10 min, trophozoite lysates were used to stimulate RAW264.7 cells (5×10^5^) in 24-well culture plates (Costar) overnight. The cells were stimulated with trophozoite lysates, LPS (1 μg/ml final conc.) (Sigma) or PBS. After incubation for 6 h, 12 h, 24 h and 48 h, culture supernatants of RAW264.7 cells were assayed for cytokines and NO production. Cells were collected and frozen for measurement of arginase activity and expression of iNOS and Arg-1 genes by qRT-PCR. Each experiment was performed at least three times.

### Determination of expression of iNOS and Arg-1 genes by qRT-PCR

Total RNA (1 μg) of treated RAW264.7 cells was purified with an RNeasy Plus Mini kit (Qiagen). cDNA was synthesized with a Primescript first-strand cDNA synthesis kit (Takara) using oligo(dT) primers. Gene expression of iNOS and Arg-1 was examined by qRT-PCR using the primers listed in Table 1. Reactions were performed as described above. Each experiment was performed at least three times.

### Measurement of NO production by Griess assay

A Griess assay was performed using 20 μl of culture supernatant of RAW264.7 cells mixed with 30 μl of distilled water and 50 μl of Griess reagent (Sigma). Absorbance was measured at 548 nm in a microplate reader [27, 28]. Experiments were performed at least three times.

### Quantification of arginase activity

Arginase activity in cell lysates was measured in RAW264.7 cells that were harvested and lysed with mammalian tissue lysis/extraction reagent (Sigma) for 15 min on a shaker and centrifuged at 13,000 g for 10 min to remove insoluble material. Sample supernatant (20 μl) was added to a well of a 96-well plate, 10 μl of substrate buffer was added, and the mixture was incubated at 37°C for 120 min for arginine hydrolysis. The reaction was stopped with 200 μl of urea in each well at room temperature for 30 min. Absorbance was measured at 430 nm in a microplate reader. One unit of arginase is the amount of enzyme that converts 1.0 mM of L-arginine to ornithine and urea per minute at pH 9.5 and 37°C. Each experiment was performed at least three times.

### Proliferation assay of RAW264.7 cells using a cell counting test

To assess whether secretory proteins from *E*. *histolytica* and *E*. *nuttalli* cause proliferation of macrophages, a cell proliferation assay was performed. RAW264.7 cells (5×10^4^) were cultured in 96-well culture plates (Costar) overnight. Cells were stimulated with 5 μl of PBS, trophozoite lysates or LPS (1 μg/ml final). CCK-8 reagent (Dojindo) was added to each well at 4 h, 10 h, 22 h or 46 h, and optical density (OD) was measured at 450 nm using a microplate reader (Bio-rad) at 6 h, 12 h, 24 h or 48 h. Each experiment was performed at least three times.

### Analysis of cytokine secretion by Luminex multiplex immunoassay

A Luminex multiplex immunoassay was performed to determine the concentrations of inflammatory cytokines using a customized Milliplex Mouse Cytokine/Chemokine Magnetic Bead Panel (Merck Millipore) for IL-1β, IL-6 and TNF-α. Briefly, 25μl of cell supernatant, control or standard was added to a 96-well plate containing 25μl of capture antibody-coated, fluorescent-coded beads. Biotinylated detection antibodies and streptavidin-PE were added to the plate after the appropriate incubation periods. After the last washing step, 150μl of sheath fluid was added to the wells, and the plate was incubated and read on a Luminex100 instrument. Five-PL regression curves were used to plot standard curves for all analytes with xPonent 3.1 software by analyzing the bead median fluorescence intensity. Results are expressed in pg/ml. Samples with quantification below the detection limit were registered as “zero” and samples above the quantification limit of the standard curve were given the value equal to the highest value of the curve. Each experiment was performed at least three times.

### Statistical analysis

Statistical analyses were performed using IBM SPSS (ver. 20, SPSS Statistics/IBM Corp., Chicago, IL, USA). qRT-PCR data were analyzed by two-tailed Mann-Whitney U test. Other data were analyzed with a two-tailed Student t-test. *P* < 0.05 was considered significant in all analyses.

## Results

### Liver tissue injury and inflammation induced by trophozoites

**Fig 1:**
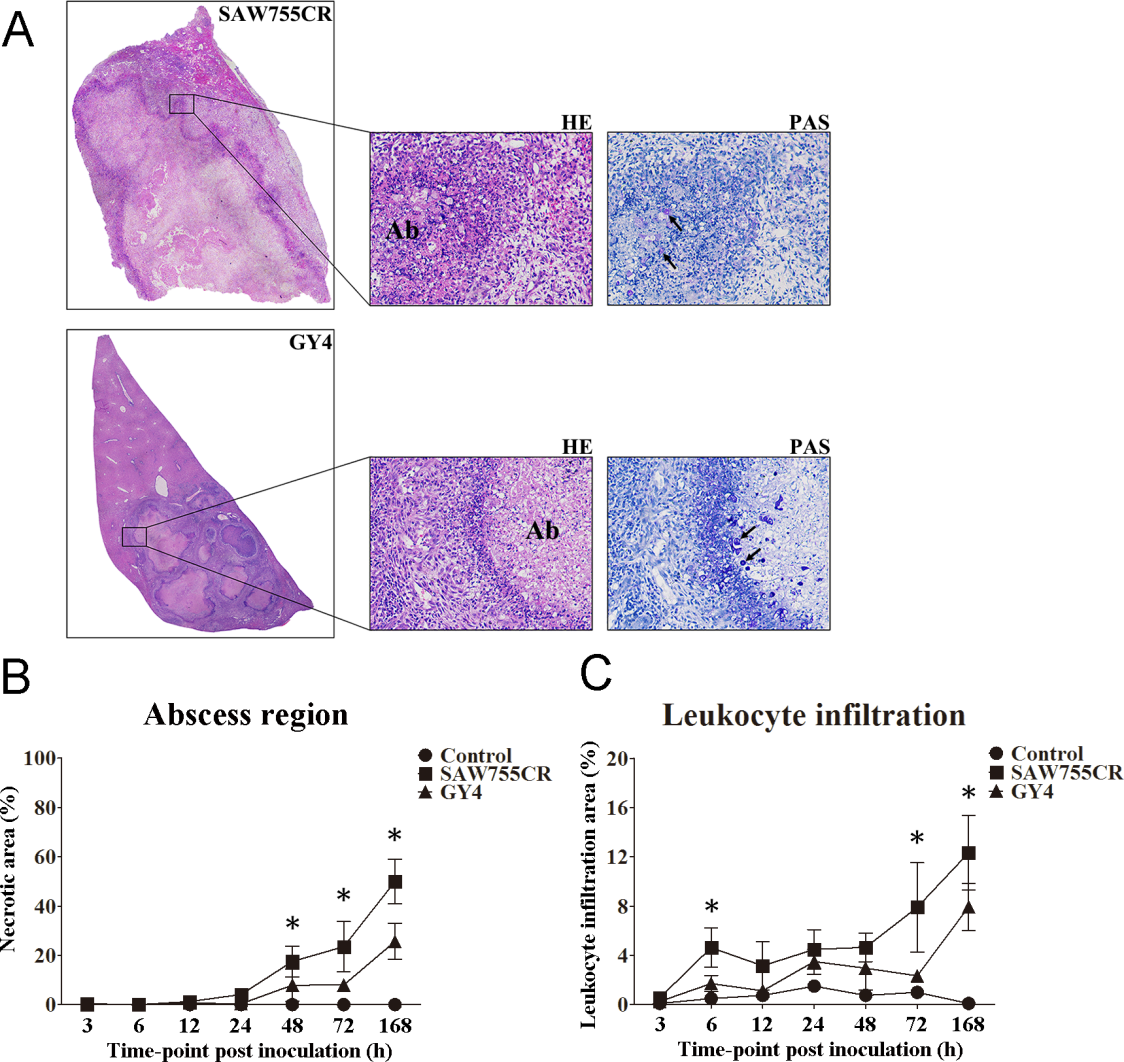
Histological features of liver in hamsters inoculated with trophozoites of *E*. *histolytica* or *E*. *nuttalli*. **(A)** Tissue section stained with hematoxylin and eosin (HE) and periodic acid-Schiff (PAS). Hamsters were inoculated with 1×10^6^ trophozoites of *E*. *histolytica* SAW755CR strain or *E*. *nuttalli* GY4 strain. Seven days after inoculation, multiple PAS-positive trophozoites (arrows) and inflammatory cells were observed in peripheral areas of necrosis (Ab). **(B,C)** Quantification of abscess region (B) and leukocyte infiltration region (C) in liver sections. Regions were evaluated in each slide of livers from hamsters (6 to 7 in each group) at different times and analyzed using Image-Pro Plus 4.5.1. Areas of interest are expressed as the percentage of the total tissue area. * P <0.05 by two-tailed Student t-test.

After inoculation of trophozoites into hamster liver, both *E*. *histolytica* SAW755 and *E*. *nuttalli* GY4 caused ALA, with a clear boundary between the abscess and normal tissue. The main area of inflammatory cell infiltration and living trophozoites was located at the edge of the abscess. At 3 h post-inoculation, inflammatory cell infiltration (mainly neutrophils) was observed (Figs 1A, S1–S3), and then infiltration of inflammatory cells increased in liver tissues. These cells were mainly monocytes and macrophages. A clear liver abscess was seen from 24 to 168 h with *E*. *histolytica* and 48 to 168 h with *E*. *nuttalli* (Fig 1B). ALA areas increased to nearly 50% with *E*. *histolytica* at 168 h, whereas the average ALA area was only 24% at 168 h with *E*. *nuttalli*. These results indicate that mild liver tissue damage was induced by *E*. *nuttalli* GY4 strain. Similarly, the area of inflammatory cell infiltration with *E*. *nuttalli* was smaller than that with *E*. *histolytica* at each time point. At 168 h, the inflammatory cell infiltration area was significantly lower with *E*. *nuttalli* (7%) than with *E*. *histolytica* (12%) (Fig 1C).

### Increased secretion of proinflammatory cytokines in liver tissues during ALA formation

**Fig 2:**
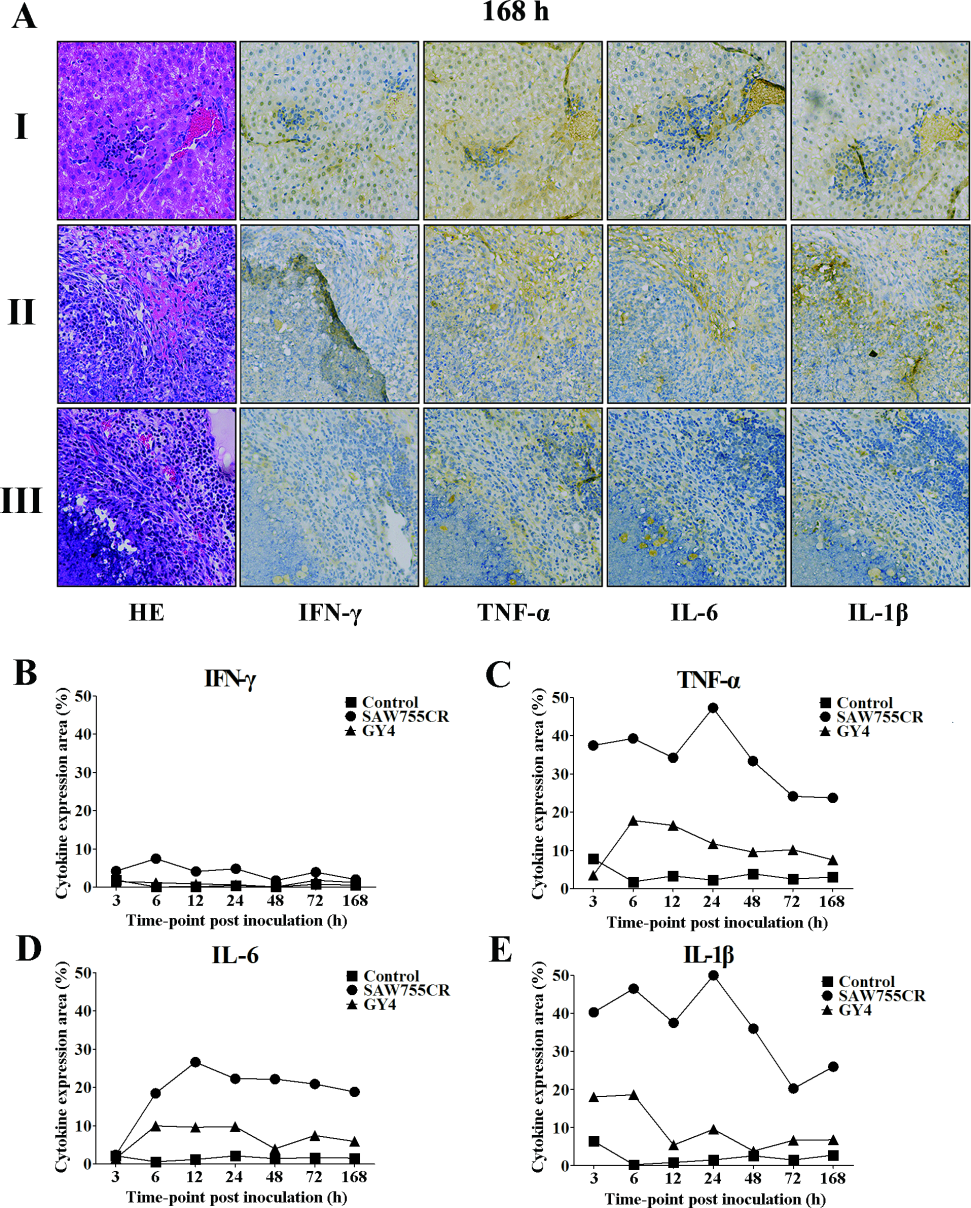
Production of IFN-γ, TNF-α, IL-1β and IL-6 in ALA of hamsters. **(A)** Immunohistochemical staining of liver sections. Liver of hamsters was inoculated with 1×10^6^ of trophozoites from *E*. *histolytica* SAW755CR strain or *E*. *nuttalli* GY4 strain. Seven days later, the liver section was stained immunohistochemically with polyclonal antibodies for IFN-γ, TNF-α, IL-1β and IL-6. I: Control group, II: SAW755CR group, III: GY4 group. **(B-E)** Cytokine scores (positive area/total image area) for IFN-γ (B), TNF-α (C), IL-1β (D) and IL-6 (E) in liver sections of hamsters at different times were determined in areas of leukocyte infiltration using Image-Pro Plus 4.5.1. Each cytokine score is the average of counting more than 50 high-power fields (×20).

To evaluate expression of cytokines during ALA formation by *E*. *nuttalli* trophozoites, hamster livers were used for immunohistochemistry at different time points, and expression levels of IFN-γ, TNF-α, IL-6 and IL-1β in liver abscesses were analyzed. The control group had low levels of these cytokines (Fig 2A) at each time point, whereas areas positive for TNF-α, IL-6 and IL-1β in liver were increased after inoculation with *E*. *histolytica* SAW755CR (Fig 2B–2E). These areas were also increased in liver inoculated with *E*. *nuttalli* GY4, but to a lesser extent compared with *E*. *histolytica* (Fig 2C–2E). These results indicate a smaller increase in expression of proinflammatory factors (TNF-α, IL-6 and IL-1β) in local tissues inoculated with *E*. *nuttalli* GY4. Little IFN-γ was detected in liver tissue after inoculation of either strain.

**Fig 3:**
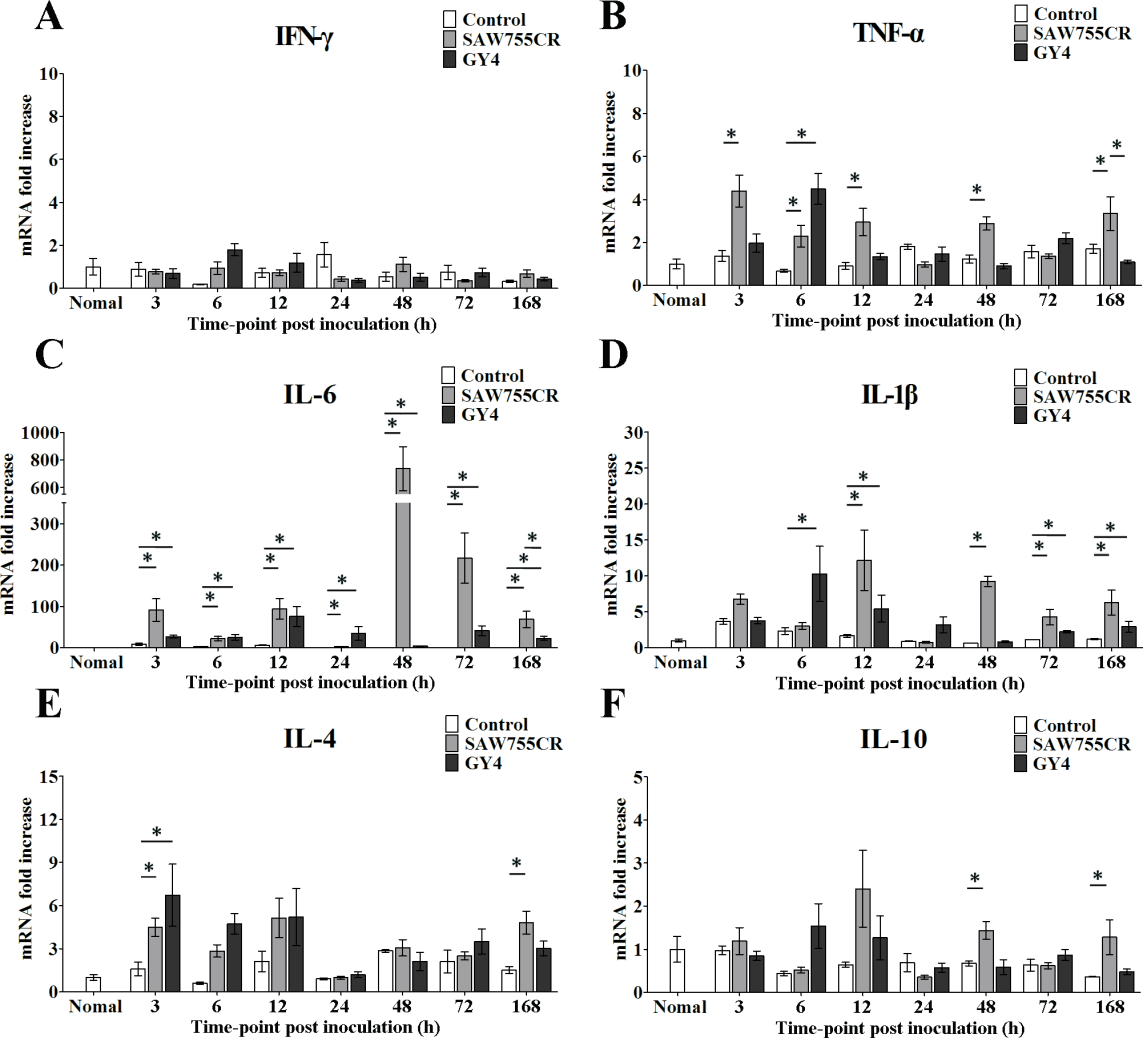
**Quantitative real-time PCR assay of gene expression of IFN-γ (A), TNF-α (B), IL-6 (C), IL-1β (D), IL-4 (E) and IL-10 (F) in ALA of hamsters.** Expression levels of cytokine genes in liver tissues from hamsters inoculated with culture medium or 1×10^6^ trophozoites of *E*. *histolytica* SAW755CR strain or *E*. *nuttalli* GY4 strain are shown as 2-ΔΔCt of the target gene relative to β-actin and normalized using the corresponding values in normal hamster. Y axes are numbers correspond to the fold increase over 1.0, assigned to normal hamster. Error bars represent standard errors of the mean calculated from three independent replicates. The abscissa shows the time post-inoculation. * P < 0.05.

To quantify the changes in cytokines during ALA formation, qRT-PCR was used to amplify IFN-γ, TNF-α, IL-6, IL-1β, IL-4 and IL-10 genes, with β-actin amplified as a reference. The results for expression levels of TNF-α, IL-6 and IL-1β in tissue at the edge of liver abscesses were similar to the immunohistochemistry data. At most time points, IL-6 and IL-1β increased significantly with *E*. *histolytica* and *E*. *nuttalli* (Fig 3). qRT-PCR showed that expression of TNF-α, IL-6 and IL-1β with *E*. *nuttalli* GY4 was lower than with *E*. *histolytica* SAW755CR at 48 h, 72 h and 168 h, and expression of IL-6 was particularly significantly lower.

### Polarization of macrophages in ALA development

**Fig 4:**
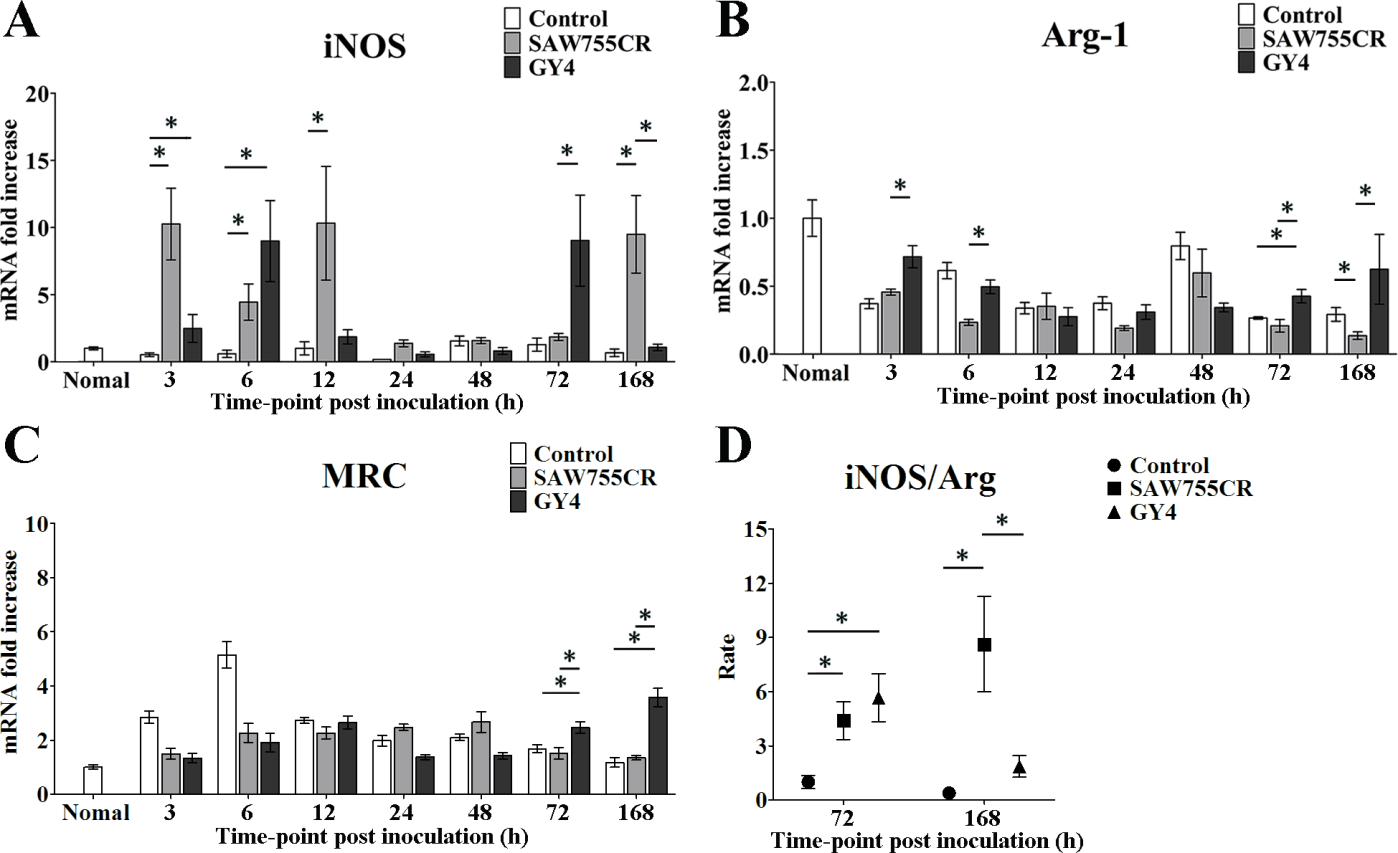
**Quantitative real-time PCR assays of expression of iNOS (A), Arg-1 (B) and MRC-I (C) and iNOS/Arg-1 mRNA ratio (D) in ALAs in hamsters.** Expression levels of cytokine genes in liver tissues from hamsters inoculated with culture medium, 1×10^6^ *E*. *histolytica* SAW755CR strain or *E*. *nuttalli* GY4 strain trophozoites are shown as 2-ΔΔCt of the target gene relative to β-actin and normalized with the corresponding values in normal hamster. Y axes in A to C are numbers correspond to the fold increase over 1.0, assigned to normal hamster. The iNOS/Arg-1 mRNA ratio in hamsters inoculated with trophozoites of *E*. *histolytica* SAW755CR or *E*. *nuttalli* GY4 is compared with that in controls at 72 h and 168 h post-inoculation. Error bars are standard errors of the mean calculated from three independent replicates. The abscissa shows the time post-inoculation. * P < 0.05.

Macrophages are immune effector cells that play an important role in ALA development. Expression of iNOS and Arg-1 genes was analyzed by qRT-PCR to examine differences in macrophage polarization in liver abscesses induced by the two species of *Entamoeba* trophozoites. Expression of iNOS rose rapidly after inoculation, whereas that of Arg-1 decreased (Fig 4A and 4B). MRC-I is a highly expressed surface receptor on M2 macrophages, and expression levels of MRC-I and Arg-1 changed similarly in qRT-PCR. In the early stage of ALA formation, MRC-I expression increased in all damaged liver tissue (including in the control group). The MRC-I level with *E*. *nuttalli* GY4 continued to increase at 72 h and 168 h, but did not rise further in the control group or with *E*. *histolytica* SAW755CR (Fig 4C). The higher iNOS/Arg-1 ratio (macrophage polarization M1/M2) at 72 h and 168 h suggests macrophage polarization toward M1 with *E*. *histolytica* SAW755CR, but toward a M1/M2 balance with *E*. *nuttalli* GY4 at 168 h (Fig 4D). These results suggest that M2 macrophages increased at 168 h after inoculation of the GY4 strain, and the milder liver tissue damage caused by *E*. *nuttalli* GY4 strain might be attributable to the increase in these macrophages.

### Expression of virulence protein genes of amebas in ALA development

**Fig 5:**
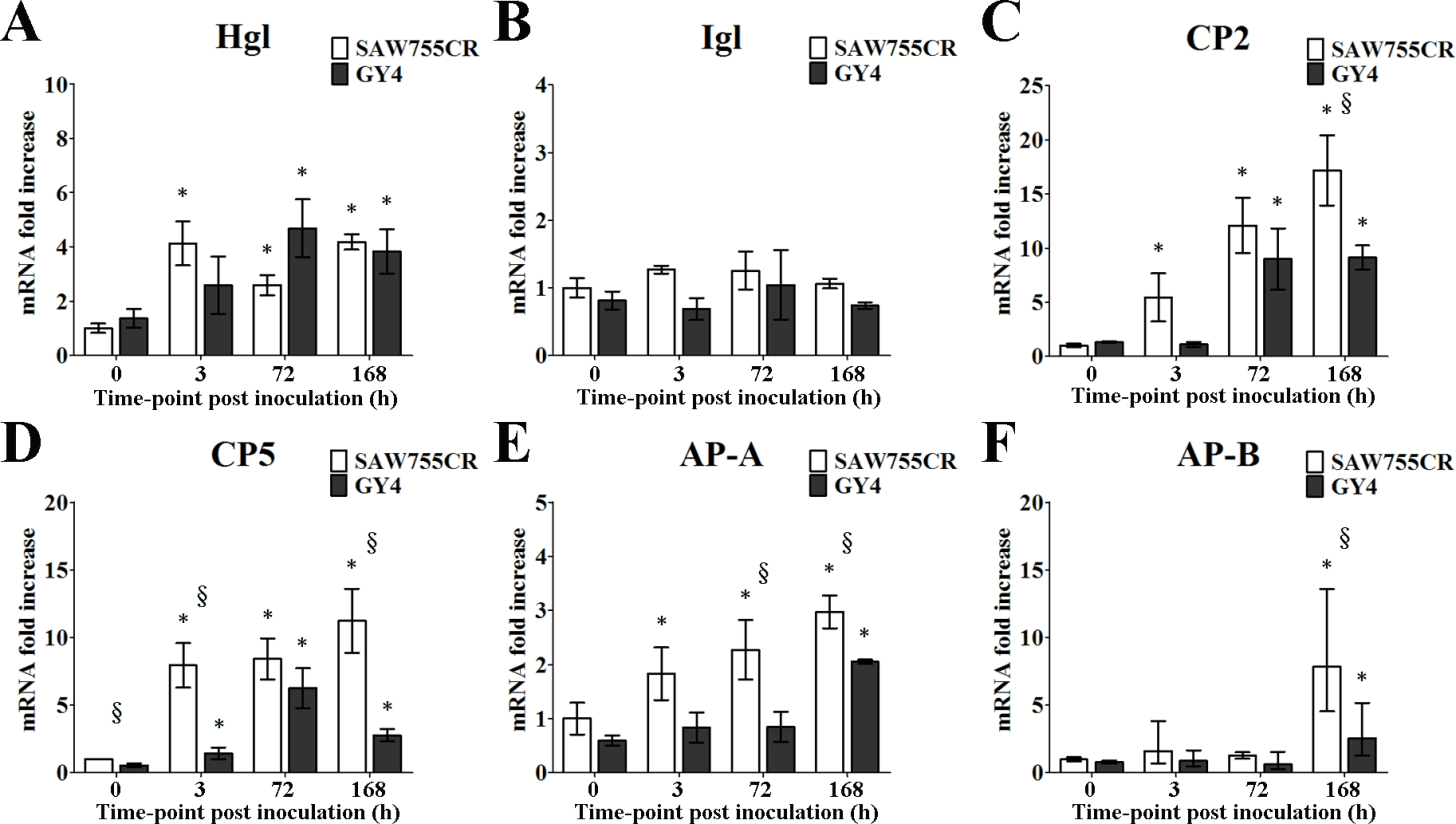
**Quantitative real-time PCR assays of expression of Hgl (A), Igl (B), CP2 (C), CP5 (D), AP-A (E) and AP-B (F) from *E*. *histolytica* or *E*. *nuttalli* in ALAs of hamsters.** Expression levels of genes for virulence factors of trophozoites in liver tissues from hamsters inoculated with *E*. *nuttalli* GY4 trophozoites are shown as 2-ΔΔCt for the target gene relative to ameba β-actin and normalized with the corresponding values from *E*. *histolytica* at 0 h, 3 h, 72 h, 168 h post-inoculation. Y axes are numbers correspond to the fold increase over 1.0, assigned to 0 h with *E*. *histolytica*. Error bars are standard errors of the mean calculated from three independent replicates. The abscissa shows the time post-inoculation. * P < 0.05 vs. 0 h, § P < 0.05 for *E*. *nuttalli* GY4 vs. *E*. *histolytica* SAW755CR.

To compare changes of virulence proteins with the two *Entamoeba* species during ALA progression, qRT-PCR was performed to examine expression levels of Hgl, Igl, CP-2, CP-5, AP-A and AP-B genes. There were significant increases in Hgl (2- to 5-fold), CP2 (5- to 16-fold), CP5 (2- to 11-fold), AP-A (2- to 3-fold) and AP-B (2- to 7-fold) after inoculation in hamster liver (Fig 5). There were few differences between the expression levels of virulence protein genes of *E*. *histolytica* and *E*. *nuttalli in vitro*; only the CP5 level was half in *E*. *nuttalli* compared to *E*. *histolytica* (Fig 5D). However, significantly lower expression of CP2, CP5, AP-A and AP-B of *E*. *nuttalli* was found at 168 h, and this lower level of virulence proteins *in vivo* may contribute to the milder liver tissue damage caused by *E*. *nuttalli* GY4.

### Polarization of RAW264.7 cells after *in vitro* stimulation with trophozoite lysates

**Fig 6:**
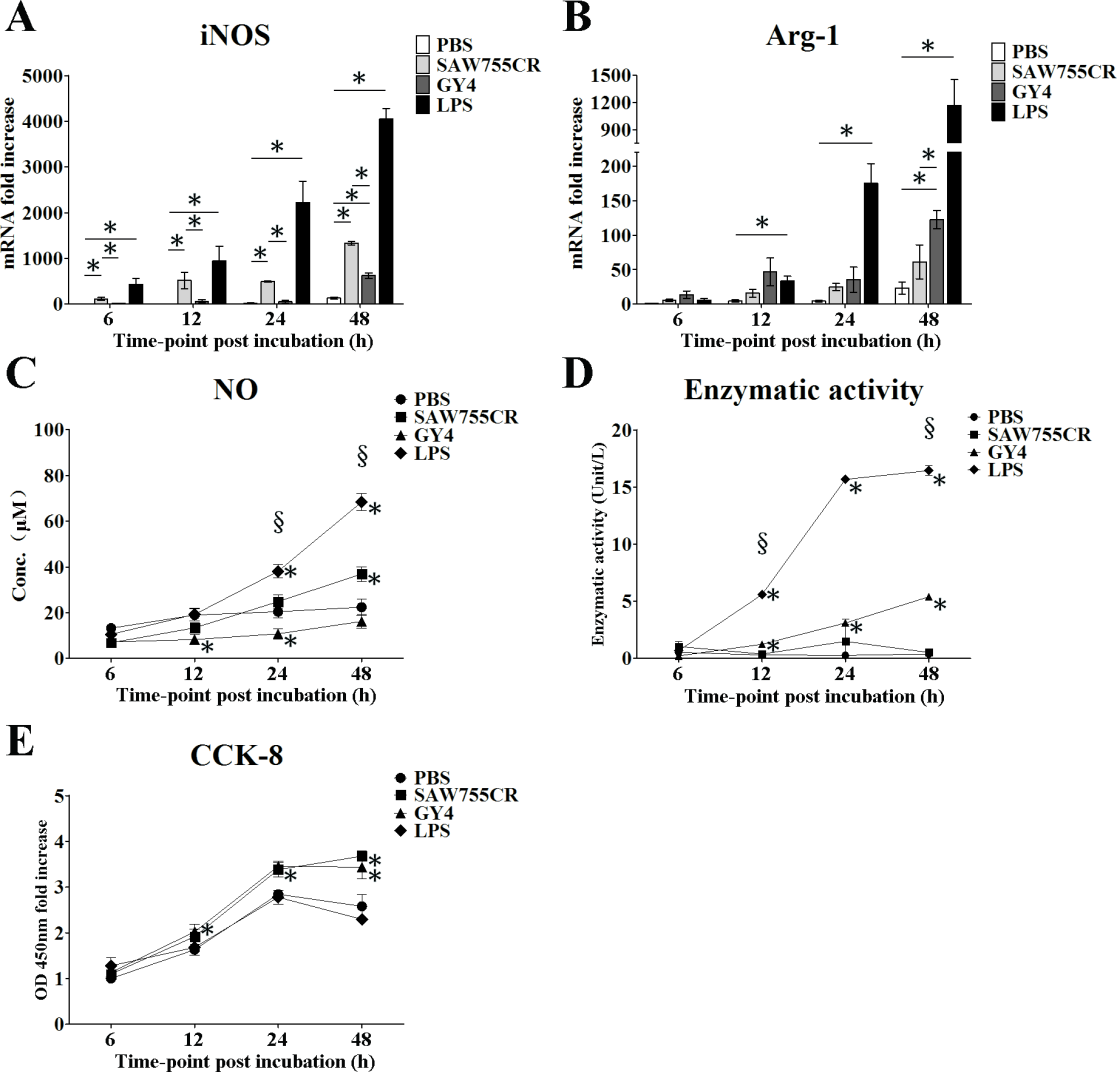
Polarization of RAW264.7 cells stimulated by trophozoite lysates *in vitro*. (A,B) Quantitative real-time PCR assays of iNOS and Arg-1 genes induced by PBS, trophozoite lysates or LPS in RAW264.7 cells. Expression levels of iNOS (A) and Arg-1 (B) are shown as 2-ΔΔCt for the target gene relative to β-actin and normalized with corresponding values in the PBS group. Y axes are numbers correspond to the fold increase over 1.0, assigned to the PBS group. * P < 0.05. (C) NO production in RAW264.7 cells. NO (μM) in culture supernatant of RAW264.7 cells stimulated by PBS, trophozoite lysates or LPS for 6 h, 12 h, 24 h and 48 h was measured by a Griess assay. (D) Arginase activity of RAW264.7 cells. Arginase activity (unit/L) was measured in RAW264.7 cell lysates stimulated by PBS, trophozoite lysates or LPS for 6 h, 12 h, 24 h and 48 h. (E) Cell proliferation assay of RAW264.7 cells. Cell proliferation was measured using a CCK-8 kit at 6 h, 12 h, 24 h and 48 h after stimulation with PBS, trophozoite lysates or LPS. The Y-axis shows the optical density (OD) of the test well minus the blank well. * P < 0.05 vs. PBS control in C to E, § P < 0.05 for *E*. *nuttalli* GY4 vs. *E*. *histolytica* SAW755CR in C and D.

To study whether secretory proteins of *Entamoeba* play an important role in polarization of macrophages, *in vitro* stimulation of mice macrophage RAW264.7 cells was performed, and expression of iNOS and Arg-1 was analyzed by qRT-PCR. The levels of both of these genes rose rapidly after stimulation with trophozoite lysates of *E*. *histolytica* or *E*. *nuttalli*. Lysate of *E*. *histolytica* induced significantly higher iNOS expression than that of *E*. *nuttalli* at 48 h (1334-fold vs. 627-fold compared to PBS). In contrast, lysate of *E*. *nuttalli* induced higher Arg-1 expression than *E*. *histolytica* (122-fold vs. 61-fold compared to PBS) (Fig 6A and 6B).

To examine the effects of secretory proteins of *Entamoeba* on NO production, RAW264.7 cells were stimulated with PBS, trophozoite lysate of *E*. *histolytica* or *E*. *nuttalli*, and LPS. A stable oxidized product of NO in the cell culture supernatants was determined by the Griess assay. Nitrate was increased by stimulation with lysate of *E*. *histolytica* at 24 h (24.8 μM) and 48 h (36.9 μM) (Fig 6C). The effect of trophozoite lysates on arginase activity was also examined. Arginase activity induced by lysates of *E*. *histolytica* and *E*. *nuttalli*, and LPS was increased at 24 h and 48 h, with lysate of *E*. *nuttalli* inducing higher arginase activity (5.4 unit/L) than that of *E*. *histolytica* at 48 h (Fig 6D). These results indicate that secretory proteins of *Entamoeba* play important roles in the polarization balance of macrophages.

A cell proliferation assay indicated that RAW264.7 cells were capable of proliferation after stimulation with trophozoite lysates of *E*. *histolytica* and *E*. *nuttalli*. An additional 33% to 42% proliferation of RAW264.7 cells occurred in comparison with PBS-stimulated cells at 48 h. There was no significant difference between lysates of *E*. *histolytica* and *E*. *nuttalli* (Fig 6E).

### Increased secretion of cytokines in RAW264.7 cells after stimulation

**Fig 7:**
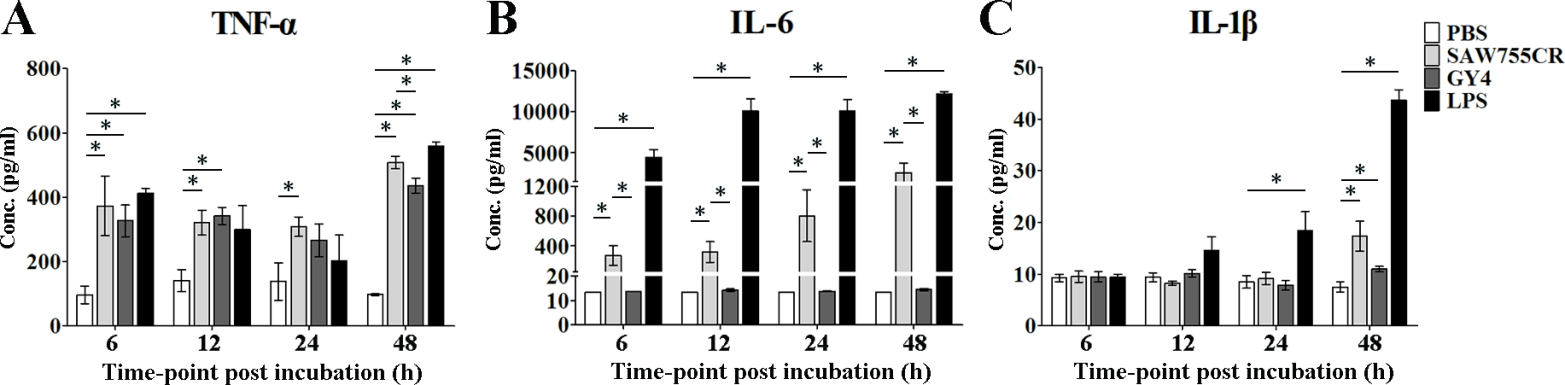
**Luminex multiplex immunoassay of TNF-α (A), IL-6 (B) and IL-1β (C) in culture supernatant of RAW264.7 cells after stimulation.** Raw264.7 cells were stimulated by PBS, trophozoite lysates or LPS for 6 h, 12 h, 24 h and 48 h. Supernatants were harvested and inflammatory cytokine concentrations (pg/ml) were measured. * P < 0.05.

Cytokine expression of RAW264.7 cells was determined using a Luminex multiplex immunoassay after stimulation with PBS, trophozoite lysate of *E*. *histolytica* or *E*. *nuttalli*, and LPS. Lysate of *E*. *histolytica* induced a significant increase in TNF-α (507.9 pg/ml), IL-6 (2573.0 pg/ml) and IL-1β (17.3 pg/ml) at 48 h. Lysate of *E*. *nuttalli* also caused a significant increase in TNF-α (435.5 pg/ml) at 48 h, but had no significant effect on IL-6 and IL-1β (Fig 7). These results are consistent with the lower expression levels of IL-6 and IL-1β with *E*. *nuttalli* GY4 in the hamster ALA model.

## Discussion

The aim of this study was to determine the histopathological features of ALAs that regulate host inflammatory immune responses following their interaction with parasites and to examine whether these features differ between *E*. *nuttalli* and *E*. *histolytica*. Our data show that both *E*. *nuttalli* and *E*. *histolytica* cause liver abscesses with a clear boundary between the abscess and normal tissue. Interestingly, lesions in hamster differed between *E*. *nuttalli* and *E*. *histolytica*, including the size of the abscess and inflammatory cell infiltration region. The ability of trophozoites to produce a liver abscess in hamsters also differs among strains of *E*. *histolytica* and *E*. *dispar*, and the SAW755 strain of *E*. *histolytica* used in this study is a highly virulent strain. Even in hamsters inoculated with *E*. *nuttalli* trophozoites, lethal cases occurred within 7 days using the NASA06 strain [10], and in hamsters inoculated with the *E*. *nuttalli* SSS212, the mean abscess size was >50% of the liver [12]. In liver tissue sections, the necrotic area with inflammatory reactions was highly extended. Therefore, the virulency of the *E*. *nuttalli* GY4 strain used in the present study may have been relatively low.

Tissue destruction during ALA formation is generally attributable to both the cytotoxicity of trophozoites and the resultant host inflammatory immune response [29]. A typical amebic lesion is characterized by a necrotic zone with edges consisting of cellular debris and inflammatory cell infiltration [30]. Such necrosis is produced by virulence factors of trophozoites, such as galactose/N-acetylgalactosamine lectin (Gal/GalNac lectin), APs and CPs [29–33]. In the present study, the expression levels of virulence factors of trophozoites in tissue was higher than that of axenically cultured trophozoites, but the levels of major CPs and APs differed between the two strains, with lower levels in *E*. *nuttalli*. Immunopathological effects also contribute to tissue destruction during liver abscess formation in the hamster model. The host inflammatory response suppresses invasive trophozoites, but also leads to severe tissue damage. During this infectious process, multiple types of inflammatory cells are recruited to the infected liver of hamsters. Infiltrating neutrophils are predominant in inflammatory regions in the initial phase of invasive liver amebic infection, followed by macrophages that accumulate rapidly during abscess formation.

Evidence from *in vivo* and *in vitro* studies suggests that macrophage-mediated anti-ameba activity is a major mode of host defense against *E*. *histolytica* infections, and has essential functions throughout ALA formation [34]. When pathogens attack, naïve macrophages can be polarized in a direction to classical activated (M1) macrophages that strongly express iNOS, which produces NO through catabolism of arginine, subsequently causing proinflammatory effects and tissue damage [35, 36]. During abscess formation, M1 macrophages release NO into infected tissue, and NO combined with toxic products from the oxidative burst then kill trophozoites. The macrophage-mediated anti-ameba activity is inhibited by arginase in a dose-dependent manner through competition with iNOS that depletes the common substrate, L-arginine [37, 38]. Macrophages can also be polarized into alternatively activated (M2) macrophages and induce Arg-1, which competes with iNOS by degrading arginine into ornithine and polyamines, giving rise to macrophages with anti-inflammatory effects and tissue repair functions [35, 36, 39]. The present study showed that *E*. *nuttalli* GY4 induced small liver abscesses at 168 h after inoculation compared with large abscesses driven by *E*. *histolytica* SAW755CR. Moreover, infiltration of inflammatory cells remained lower in abscess lesions of *E*. *nuttalli* compared to those caused by *E*. *histolytica*.

In amebic liver lesions, secretion of Gal/GalNac lectin, APs and CPs by trophozoites also results in destruction of neutrophils and liberation of their toxic products, which may play an important role in enlargement of abscess lesions [29, 30]. Immunosuppressive and tissue repair functions play critical roles in control of inflammation by producing anti-inflammatory mediators [36, 39]. In this study, both *Entamoeba* species caused increased levels of iNOS in liver lesions of hamsters and decreased arginine at the early stage of ALA formation, indicating elevation of M1 macrophages, which are involved in host defense and tissue damage. Significantly, the level of arginine increased with *E*. *nuttalli* at 168 h after trophozoite inoculation, which suggests greater elevation of M2 macrophages compared with *E*. *histolytica* infection. The increased proportion of M2 macrophages in liver abscess lesions might attenuate tissue damage through accelerated tissue repair, and this might explain the smaller abscesses and milder liver tissue damage in the animal model infected with *E*. *nuttalli*.

There are several key factors in macrophage polarization during infection, with pathogens and their virulence proteins being the fundamental regulators [39–41]. The current study indicated that proteins secreted by both *Entamoeba* species were able to induce macrophage polarization and skew differentiation towards M1 or M2 phenotypes. With *E*. *histolytica*, the macrophage polarization skewed towards the M1 phenotype, as shown by the significant increase in iNOS expression and multiple proinflammatory cytokines, such as TNF-α, IL-1 and IL-6, exerting immunoregulatory roles during infection. With *E*. *nuttalli*, the polarization trend of macrophages was not as clear, based on the lower levels of iNOS and cytokines and higher production of arginine, compared to *E*. *histolytica* infection. These results suggest an equilibrium in macrophage polarization. Several studies have shown amebicidal activity of macrophages mediated by iNOS mRNA expression and NO production [30, 42, 43]. There is also evidence of direct macrophage activity by amebic virulence factors, and *E*. *nuttalli* secreted fewer virulence factors than *E*. *histolytica* based on protein profiles. Taken together, these data indicate that virulence factors inducing macrophage polarization in hamster liver lesions switch to a protective M2 phenotype from a destructive M1 phenotype, leading to decreased NO production, which reduces immunopathological tissue damage.

T-cell cytokine responses can be divided into different classes based on the combination of cytokines produced. Th1 cells secrete cytokines including IL-2, IFN-γ and TNF-β that promote differentiation and activity of macrophages and cytotoxic T cells, and lead primarily to a cytotoxic immune response. In contrast, the Th2 cytokine response is characterized by IL-4, IL-5, IL-6, IL-9 and IL-10 production [44]. These cytokines, the levels of which correlate with the degree of tissue damage, are released by attacked host cells or effector cells. IFN-γ is a suppressive cytokine that can clear the parasite [45]. In the present study, there was no significant increase in IFN-γ during ALA development in hamsters, perhaps suggesting that persistent progression of lesions facilitates invasive amebiasis. In contrast, TNF-α and IL-6, which are inflammatory factors, were strongly sustained and expressed during progression of tissue damage. Macrophages were clearly the major effector cells secreting these cytokine mediators. M1 macrophages secreted proinflammatory cytokines, including TNF-α, IL-1β and IL-6, which activate phagocytes to kill pathogens, but also cause tissue damage [46, 47]. The results of immunohistochemistry and qRT-PCR indicated that the levels of multiple cytokines increased during ALA formation. These results suggest that macrophage polarization might profoundly affect the degree of tissue damage in ALA formation.

At the early stage of infection, TNF-α, IL-1β and IL-6 showed an increasing trend with both *Entamoeba* species, but with higher levels with *E*. *histolytica* than with *E*. *nuttalli*. The increased TNF-α and IL-6 during amebic tissue damage results in activation of macrophages to release NO and thereby exert an anti-inflammatory effect. Our data show that macrophage polarization by *E*. *histolytica* SAW755CR induced greater upregulation of iNOS expression at the transcriptional level, resulting in a higher proportion of M1 polarized macrophages, which then secreted higher levels of proinflammatory cytokines and aggravated amebic tissue damage. The released TNF-α and IL-1β feedback to further skew macrophage differentiation towards the M1 phenotype. Consequently, small multiple abscesses merge with each other and coalesce to form large single abscesses after infection with *E*. *histolytica* SAW755CR. M2 macrophages generally have high levels of mannose receptors and scavenger receptors, and play important roles in polarized Th2 reactions. For example, M2 macrophages promote the encapsulation and killing of parasites and have immunoregulatory and anti-inflammatory functions [48]. This macrophage population is also thought to play a critical role in negative regulation of host protective immunity against microbial infections. Thus, M2 macrophages modulate expression of anti-inflammatory cytokines such as MRC or transforming growth factor, and thereby modulate suppression of tissue inflammation and enhance tissue repair [49]. In *E*. *nuttalli* infection, expression levels of TNF-α and IL-1β decreased at 168 h after inoculation, whereas expression of MRC was upregulated with the same trend as that of Arg-1. The downregulation of TNF-α and IL-1β suggests that tissue damage might be slowed in *E*. *nuttalli* infection. Macrophage polarization tends to reach an equilibrium with the increase in the M2 phenotype. Repair-associated factors begin to take effect and inhibit ALA formation and development. Finally, this leads to formation of multiple small abscesses that are incapable of coalescing into larger lesions, consistent with the finding that *E*. *nuttalli* GY4 forms smaller liver abscesses than *E*. *histolytica* SAW755. Thus, our results show that differential secretion of amebic virulence factors in *E*. *nuttalli* infection may trigger lower cytokine secretion and promote polarization of macrophages towards a M1/M2 balance.

In consideration of intestinal immunity that is the first line of defense against amoeba trophozoites, a critical aspect in *Entamoeba* pathogenesis is to overcome the colonic epithelial barrier [50–53]. The intestinal bacterial microbiota is another important factor that influence the pathogenesis of *E*. *histolytica*. This could be interrelated to direct ingestion of intestinal bacteria that increase the expression of virulence proteins of *E*. *histolytica* [54–58]. The bacteria could also alter the immune status of host intestine to prevent or promote amoebiasis. For instance, the increasing of IL-23, IL-17, dendritic cells and neutrophil induced by segmented filamentous bacterium in the cecum mediated protection from *E*. *histolytica* [59, 60]. *E*. *nuttalli* trophozoites may elicit ALA formation with intense inflammatory reaction in human if the parasites translocate to liver. However, *E*. *nuttalli* is probably not adapted to intestinal microenvironment of human and unable to invade beyond the colonic epithelial barrier of human under natural conditions.

There is unambiguous evidence of an intense acute inflammatory reaction in hamster liver in infection by both *Entamoeba* species, but no evidence showing that these events in hamster liver also occur in human liver. Moreover, it is unknown whether *E*. *nuttalli* trophozoites produce intestinal ulcer in human and non-human primates. Additionally, as well known in genetic restriction, a T-cell receptor recognizes a particular antigenic peptide presented by a specific histocompatibility complex (MHC) molecule, and this interaction is associated with susceptibility or resistance to pathogen infection [61–64]. For instance, highly polymorphic HLA genes have an enormous capacity to bind to viral peptides associated with HBV infection [65] and a single MHC supertype confers qualitative resistance to *Plasmodium relictum* infections in avian malaria [66]. Consequently, host MHC molecule may also play a key role in determination of host susceptibility to *E*. *nuttalli*.

In conclusion, histopathological features and expression levels of proinflamatory factors in ALAs formed by *E*. *nuttalli* were identified in this study. The results also suggest that the difference of tissue damage in infection by *E*. *histolytica* and *E*. *nuttalli* is due to the levels of secretion of various cytokines, regardless of the extent of macrophage polarization. In any event, both *Entamoeba* species induced intense acute inflammatory reactions in liver of hamsters after infection.

## Supporting information

S1 Fig **Immunohistochemical staining of liver sections 3 h (A) or 6 h (B) after intrahepatic inoculation.** Liver of hamsters was inoculated with 1×10^6^ of trophozoites from *E*. *histolytica* SAW755CR strain or *E*. *nuttalli* GY4 strain. 3 h or 6 h later, the liver section was stained immunohistochemically with polyclonal antibodies for IFN-γ, TNF-α, IL-1β and IL-6. I: Control group, II: SAW755CR group, III: GY4 group.
(PDF)

S2 Fig **Immunohistochemical staining of liver sections 12 h (A) or 24 h (B) after intrahepatic inoculation.** Liver of hamsters was inoculated with 1×10^6^ of trophozoites from *E*. *histolytica* SAW755CR strain or *E*. *nuttalli* GY4 strain. 12 h or 24 h later, the liver section was stained immunohistochemically with polyclonal antibodies for IFN-γ, TNF-α, IL-1β and IL-6. I: Control group, II: SAW755CR group, III: GY4 group.
(PDF)

S3 Fig **Immunohistochemical staining of liver sections 48 h (A) or 72 h (B) after intrahepatic inoculation.** Liver of hamsters was inoculated with 1×10^6^ of trophozoites from *E*. *histolytica* SAW755CR strain or *E*. *nuttalli* GY4 strain. 48 h or 72 h later, the liver section was stained immunohistochemically with polyclonal antibodies for IFN-γ, TNF-α, IL-1β and IL-6. I: Control group, II: SAW755CR group, III: GY4 group.
(PDF)

S1 Text The nucleotide sequences of virulence protein genes of *E*. *nuttalli* GY4 strain. (DOCX)
