# Supplementary figures and images for: Characteristics of inflammatory reactions during development of liver abscess in hamsters inoculated with Entamoeba nuttalli
Source: PLoS Negl Trop Dis. 2018 Feb 8;12(2):e0006216. doi: 10.1371/journal.pntd.0006216 (PMC5821383; doi:10.1371/journal.pntd.0006216)

**A****3h****I**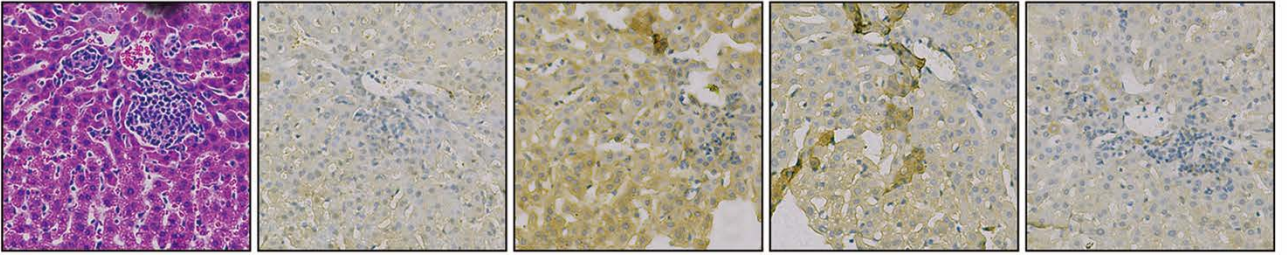**II**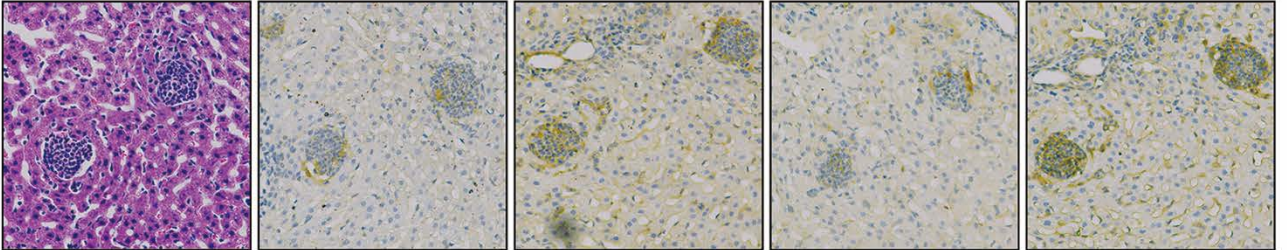**III**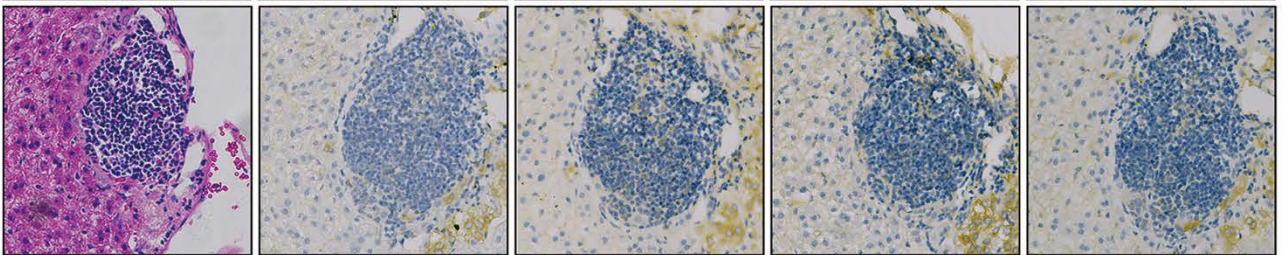**HE****IFN- $\gamma$** **TNF- $\alpha$** **IL-6****IL-1 $\beta$** **B****6h****I**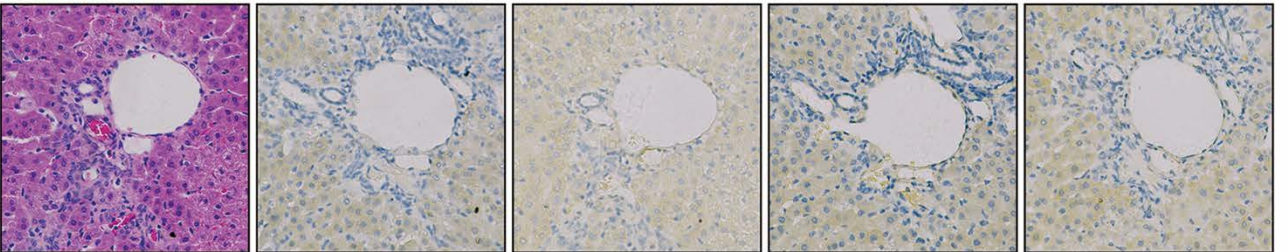**II**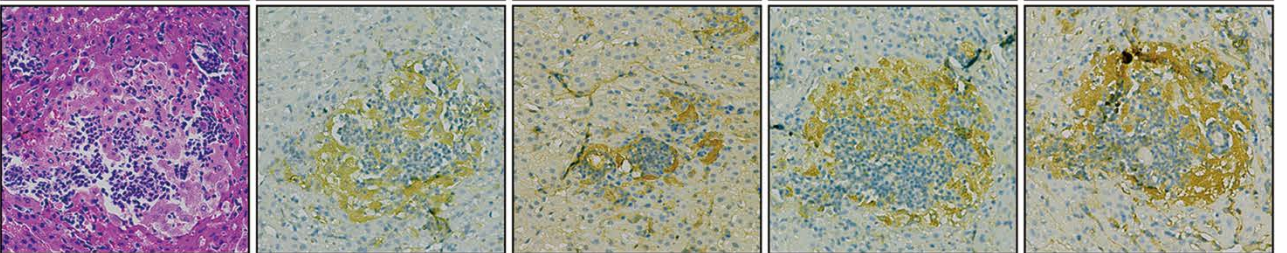**III**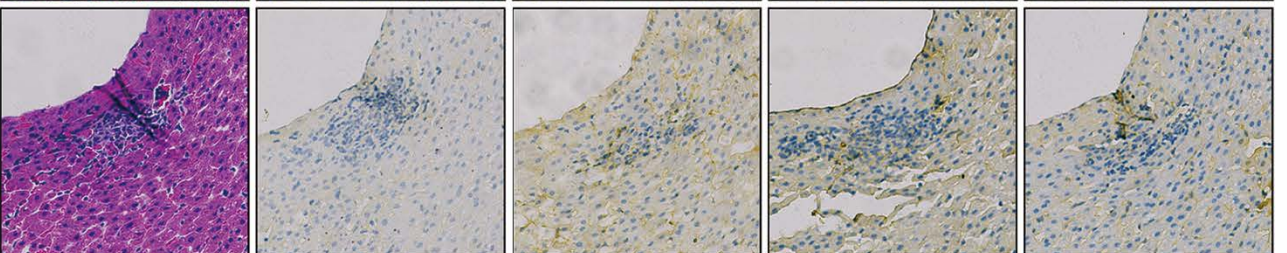**HE****IFN- $\gamma$** **TNF- $\alpha$** **IL-6****IL-1 $\beta$**

Supplement: S1 Fig — Immunohistochemical staining of liver sections 3 h (A) or 6 h (B) after intrahepatic inoculation. Liver of hamsters was inoculated with 1×10^6^ of trophozoites from E. histolytica SAW755CR strain or E. nuttalli GY4 strain. 3 h or 6 h later, the liver section was stained immunohistochemically with polyclonal antibodies for IFN-γ, TNF-α, IL-1β and IL-6. I: Control group, II: SAW755CR group, III: GY4 group. (PDF) [file pntd.0006216.s001.pdf]

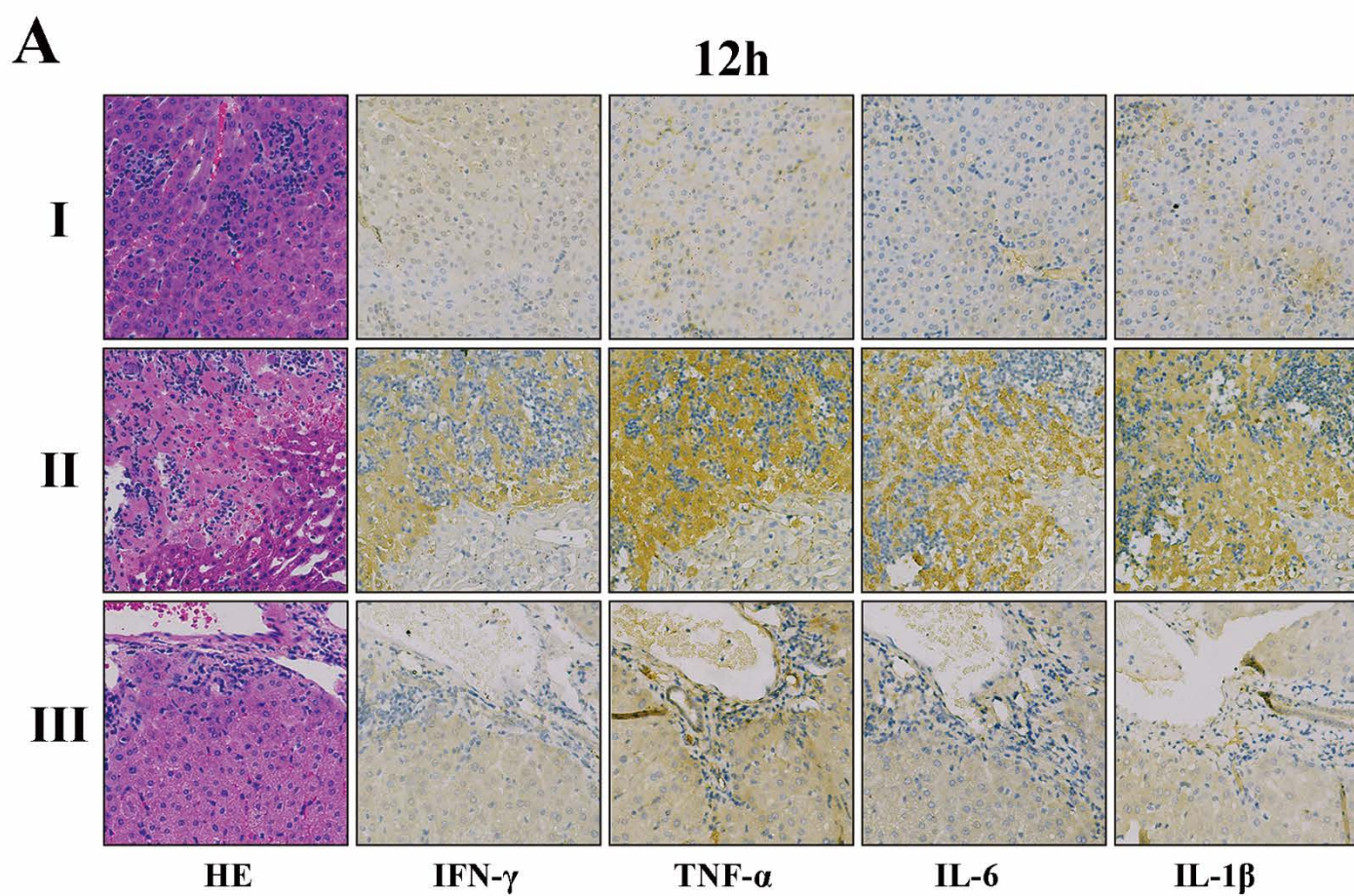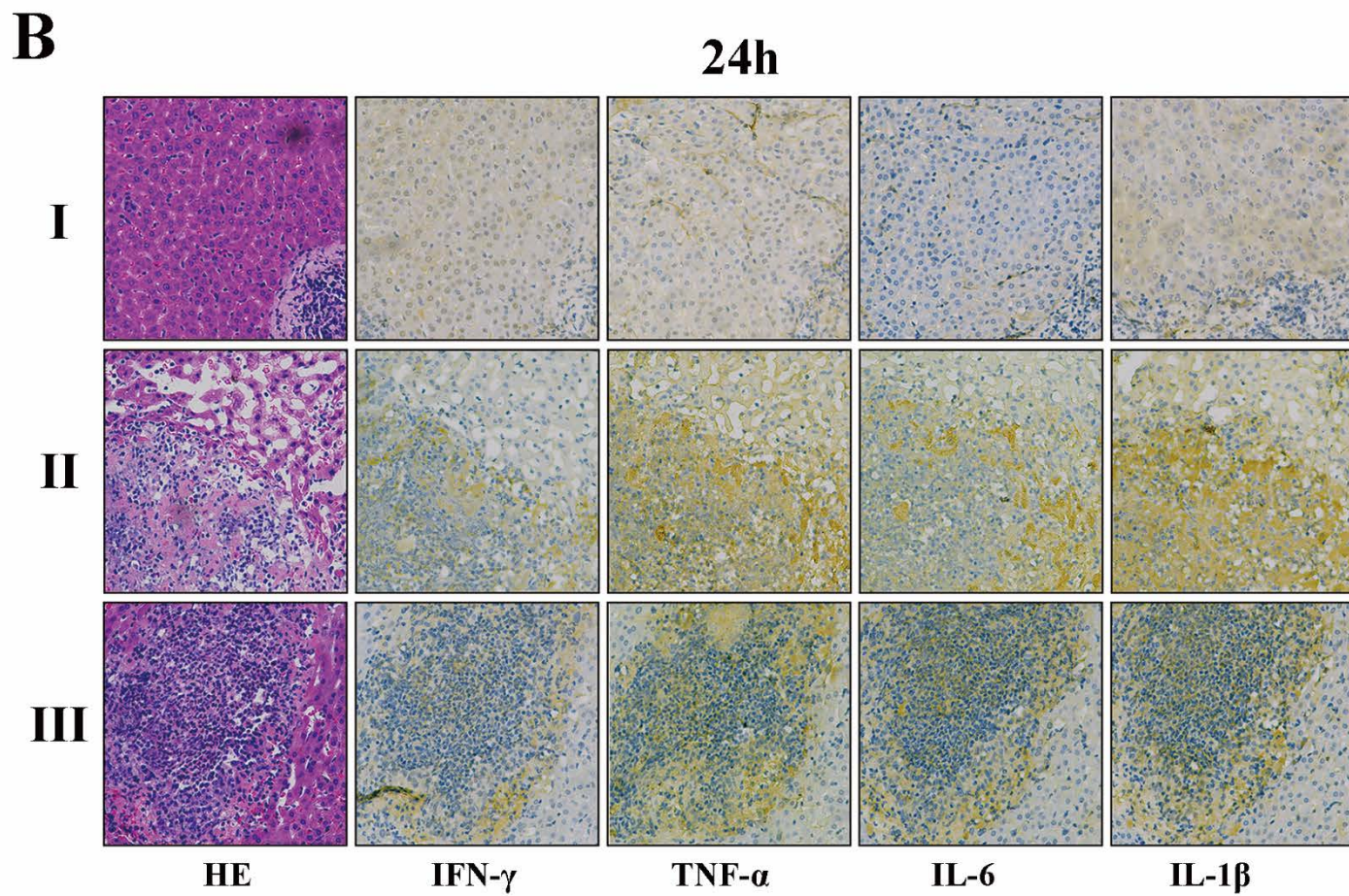

Supplement: S2 Fig — Immunohistochemical staining of liver sections 12 h (A) or 24 h (B) after intrahepatic inoculation. Liver of hamsters was inoculated with 1×10^6^ of trophozoites from E. histolytica SAW755CR strain or E. nuttalli GY4 strain. 12 h or 24 h later, the liver section was stained immunohistochemically with polyclonal antibodies for IFN-γ, TNF-α, IL-1β and IL-6. I: Control group, II: SAW755CR group, III: GY4 group. (PDF) [file pntd.0006216.s002.pdf]

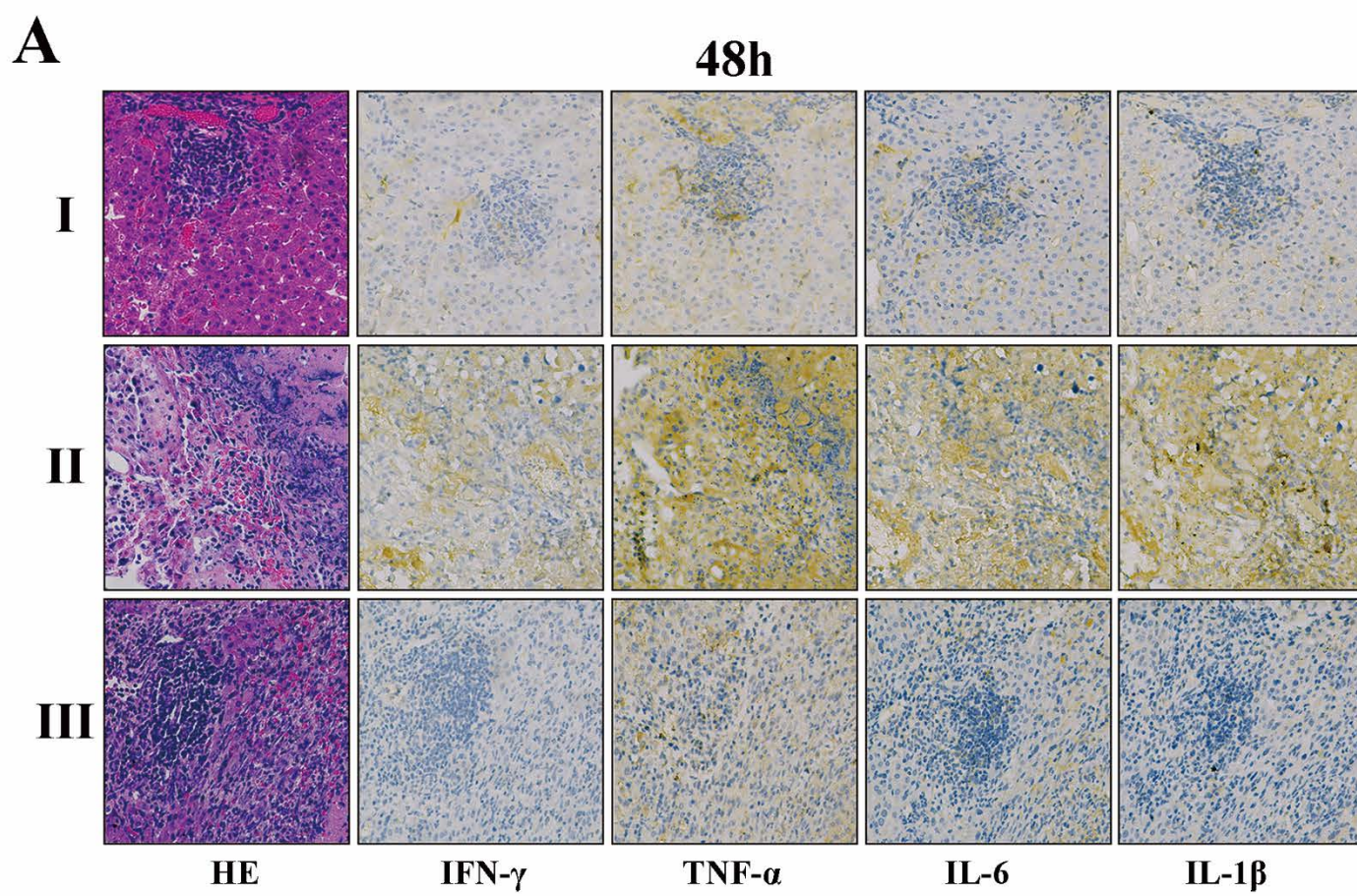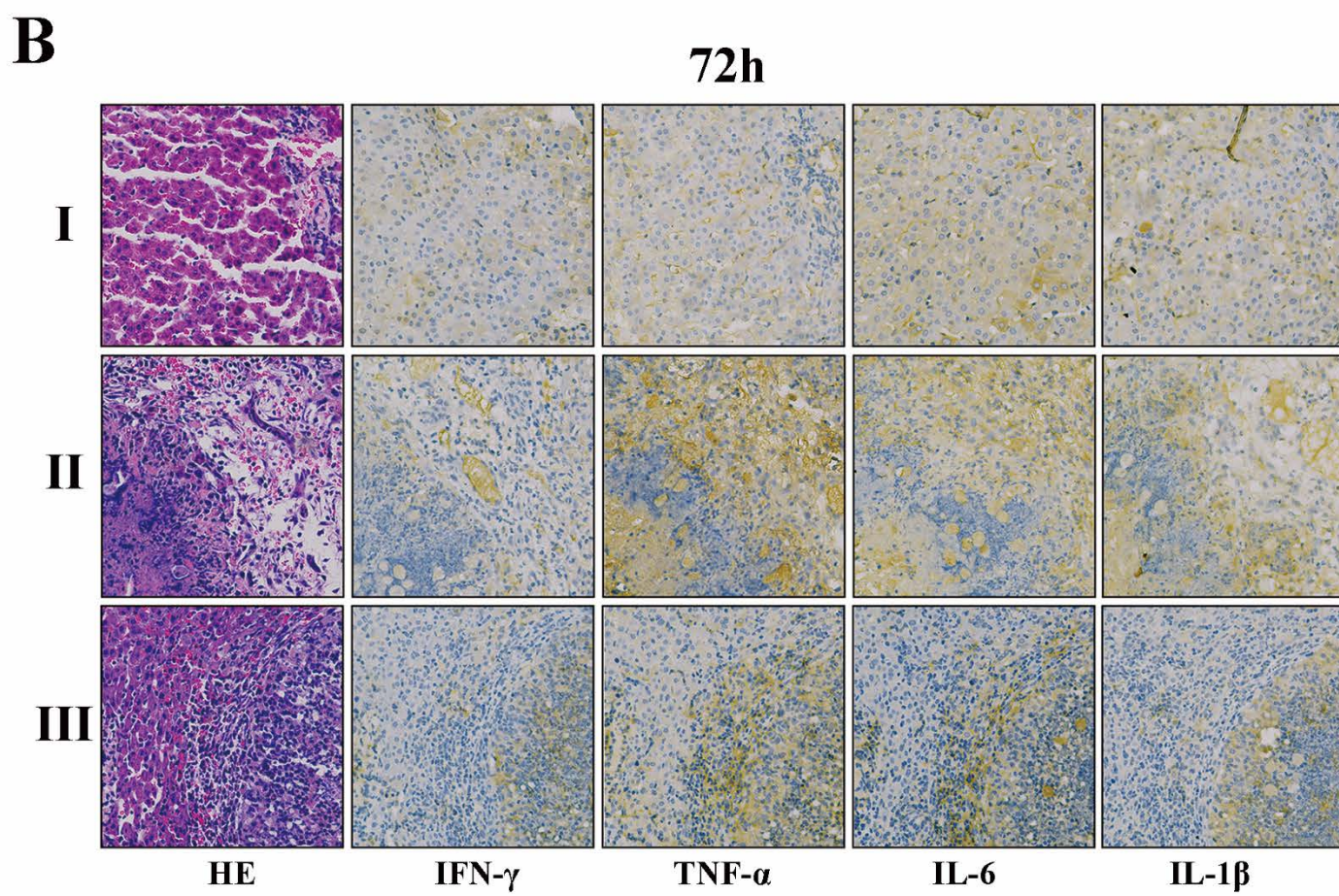

Supplement: S3 Fig — Immunohistochemical staining of liver sections 48 h (A) or 72 h (B) after intrahepatic inoculation. Liver of hamsters was inoculated with 1×10^6^ of trophozoites from E. histolytica SAW755CR strain or E. nuttalli GY4 strain. 48 h or 72 h later, the liver section was stained immunohistochemically with polyclonal antibodies for IFN-γ, TNF-α, IL-1β and IL-6. I: Control group, II: SAW755CR group, III: GY4 group. (PDF) [file pntd.0006216.s003.pdf]
