# Supplementary material for: Characteristics of inflammatory reactions during development of liver abscess in hamsters inoculated with Entamoeba nuttalli
Source: PLoS Negl Trop Dis. 2018 Feb 8;12(2):e0006216. doi: 10.1371/journal.pntd.0006216 (PMC5821383; doi:10.1371/journal.pntd.0006216)
Supplement: S1 Text — (DOCX) [file pntd.0006216.s004.docx]

>*Entamoeba nuttalli* GY4 strain-Hgl-clone1

CACATATTCATCATGTGGTGGAGATTCTACAGGATCAGTATGTAAATGTGATGCATCAACTGGTAATAAATGTCAATGTAATAAAGTACAAAACGGTAATTATTGTGATTCTAGTAAACATCAAATTTGTGATTATACTGGAGATAAACCAAAATGTATTGTCTCTGAATGTACAGAAGATCTTGTTAGAGATGGATGTCTTATTAAGAGATGTAATAAGACAAGTAAAACAACATATTGGGAAAATGTTGATTGTTCTAAAACTAAGATTGAATTTGCTAAAGATGGCAGATCTGAAACTATGTGTAAGCAATATTATTCAACTACATGTTTGAATGGACAATGTGTTGTTCAAGCAGTTGGTGATGTTTCTAATGTAGGATGTGGATATTGTTCAATGGGAACAAATAATGTTATTACATATCATGATGATTGTAATTCACGTAAATCAC

>*Entamoeba nuttalli* GY4 strain-Hgl-clone2

CACATATTCATCATGTGGTGGAGATTCTACAGGATCAGTATGTAAATGTGATTCTACAACAAAGAATCAATGTCAATGTACTCAAGTAAAAAATGGTAATTATTGTAATTCTAACAACCATGAAATTTGTGATTATACAGGAGATAAACCAAAATGTAAAGTGTCTAATTGCACAGAAGATCTTGTTAGAGATGGATGTCTTATTAAGAGATGTAATGAAACAAGTAAAACAACATATTGGGAAAATCTTGATTGTTCTAAAACTGAAGTTAAATTTGCTAAAGATGATAAATCTGAAACTATGTGTAAGCCATATTATTCAGCTACATGTTTGAATGGACAATGTGTTGTTCAAGCAGTTGGTGATGTTTCTAATGTAGGATGTGGATATTGTTCAATGGGAACAAATAATGTTATTACATATCATGATGATTGTAATTCACGTAAATCAC

>*Entamoeba nuttalli* GY4 strain-Hgl-clone3

TACATATTCATCATGTGGTGGAGATTCTACAGGTTCAGTATGTAAATGTGATGCATCAACTGGTAATAAATGTCAATGTAATAAGGTAGAAAATGGTAATTATTGTGATTCTAGTAAACATGAGATTTGTGATTATACTGGAGATAAACCAAAATGTATTGTTTCTGATTGTACAGAAGATCTTGTTAGAGATGGATGTCTTATTAAGAGATGTAATAAGACAAGTAAAACAACATATTGGGAGAATGTTGATTGTTCAAACACTAAGATTGAATTTGCTAAAGATGGTAAATCTGAAACTATGTGTAAGCCATATTATTCAGCTACATGTTTGAATGGACAATGTGTTGTTCAAGCAGTTGGTGATGTTTCTAATGTAGGATGTGGATATTGTTCAATGGGAACAGACAATATTATTAAATATCATGATGATTGTGATTCACGTAAATCAC

>*Entamoeba nuttalli* GY4 strain-AP-A

CATCGTCTTTGTTTTAATCTTCGCTGTTGCTTTTGCAGTTACTGCTACTCATCAAGGAGAAATCATCTGCAATCTTTGCACTGGACTTATTAATACACTTGAAAACCTCCTTACCACTAAGGGAGCTGACAAAGTAAAAGATTATATTGGCAGCCTTTGCAACAAAGCTTCAGGATTCATTTCTACTCTTTGCACCAAGGTTCTTGATTTTGGAATTGATAAACTCATCCAACTTATTGAAGACAAAGTTGATGCCAATGCTATTTGTGCTAAGATCATGCTTGCA

>*Entamoeba nuttalli* GY4 strain-AP-B

TATGAGAGCTATTATTTTTGTTTTAATCTTTGCTATTGCCTTTGCTGCAACAAGAGAAGGAGCTATTCTTTGCAATCTTTGTAAAGATACAGTTAAGCTCGTTGAAAATCTTTTAACTGTTGATGGTGCACAAGCTGTTAGACAATATATCGACAACCTTTGTGGTAAAGCTAATGGATTCCTTTCAACCCTTTGTGAAAAAATTCTTTCATTTGGTGTTGATGAACTTGTCAAGCTTATTGAAAATCACGTAGATCCAGTCGTTGTTTGCGAAAAGATTCCGGCTTGT

>*Entamoeba nuttalli* GY4 strain-CP2

TTACTTGCTATTGCAAGTGCCATTGATTTCAATACATGGGCTGCTAAAAACAATAAACACTTCACAGCAATTGAAAAGCTTAGAAGAAGAGCTATTTTCAATATGAATGCTAAATTTGTTGATAGTTTCAATAAAATTGGCTCATTCAAATTATCTGTAGAAGGACCATTTGCTGCTATGACTAATGAAGAATATAGAACTCTTCTTAAATCTAAAAGAACTGCTGAAGAAAAAGGAGAAGTTAAATATTTGAATATCCAAGCACCAGAATCAGTAGATTGGAGAAAAGAAGGAAAAGTAACCCCAATTAGAGATCAAGCACAATGTGGATCATGCTATACATTTGGTTCACTTGCAGCTCTTGAAGGAAGATTATTAATTGAAAAAGGAGGTAATGCTAATACACTTGATCTTTCAGAAGAACATATGGTTCAAtGCACAAGAAATAAGGGAAATAATGGATGTAATGGAAGGACTTG

>*Entamoeba nuttalli* GY4 strain-CP5

TTTGACCAGGAAGGAAAAAGCACTTATGCGTGCTGCTGCTGAAGGACCAGTTGCTGCTGCTATAGATGCTTCAGGAGTTAAATTCCAATTATATAAGAGTGGTATATATAATAGTAAAGAATGTTCATCAACTCAGCTTAATCATGGTGTAGCAGTAGTTGGTTATGGTACTCAAAATGGAACTGAATATTGGATTGTTAGAAATTCATGGGGAACTATTTGGGGAGATCAAGGATATGTTTTAATGTCAAGAAATAAGAATAATCAATGTGGTATTGCTTCAGGAGCTGCTTATCCAGTTGGGGTTGCTGATGCT
